# Dilated cardiomyopathy mutations in thin-filament regulatory proteins reduce contractility, suppress systolic Ca^2+^, and activate NFAT and Akt signaling

**DOI:** 10.1152/ajpheart.00272.2020

**Published:** 2020-07-03

**Authors:** Paul Robinson, Alexander J. Sparrow, Suketu Patel, Marta Malinowska, Svetlana N. Reilly, Yin-Hua Zhang, Barbara Casadei, Hugh Watkins, Charles Redwood

**Affiliations:** ^1^Division of Cardiovascular Medicine, Radcliffe Department of Medicine, University of Oxford, Oxford, United Kingdom; ^2^British Heart Foundation, Centre of Research Excellence, University of Oxford, Oxford, United Kingdom

**Keywords:** Akt, calcium, cardiomyopathy, NFAT transcription factor, RAC-α serine/threonine-protein kinase, tropomyosin, troponin

## Abstract

Dilated cardiomyopathy (DCM) is clinically characterized by dilated ventricular cavities and reduced ejection fraction, leading to heart failure and increased thromboembolic risk. Mutations in thin-filament regulatory proteins can cause DCM and have been shown in vitro to reduce contractility and myofilament Ca^2+^-affinity. In this work we have studied the functional consequences of mutations in cardiac troponin T (R131W), cardiac troponin I (K36Q) and α-tropomyosin (E40K) using adenovirally transduced isolated guinea pig left ventricular cardiomyocytes. We find significantly reduced fractional shortening with reduced systolic Ca^2+^. Contraction and Ca^2+^ reuptake times were slowed, which contrast with some findings in murine models of myofilament Ca^2+^ desensitization. We also observe increased sarcoplasmic reticulum (SR) Ca^2+^ load and smaller fractional SR Ca^2+^ release. This corresponds to a reduction in SR Ca^2+^-ATPase activity and increase in sodium-calcium exchanger activity. We also observe dephosphorylation and nuclear translocation of the nuclear factor of activated T cells (NFAT), with concordant RAC-α-serine/threonine protein kinase (Akt) phosphorylation but no change to extracellular signal‐regulated kinase activation in chronically paced cardiomyocytes expressing DCM mutations. These changes in Ca^2+^ handling and signaling are common to all three mutations, indicating an analogous pathway of disease pathogenesis in thin-filament sarcomeric DCM. Previous work has shown that changes to myofilament Ca^2+^ sensitivity caused by DCM mutations are qualitatively opposite from hypertrophic cardiomyopathy (HCM) mutations in the same genes. However, we find several common pathways such as increased relaxation times and NFAT activation that are also hallmarks of HCM. This suggests more complex intracellular signaling underpinning DCM, driven by the primary mutation.

**NEW & NOTEWORTHY** Dilated cardiomyopathy (DCM) is a frequently occurring cardiac disorder with a degree of genetic inheritance. We have found that DCM mutations in proteins that regulate the contractile machinery cause alterations to contraction, calcium-handling, and some new signaling pathways that provide stimuli for disease development. We have used guinea pig cells that recapitulate human calcium-handling and introduced the mutations using adenovirus gene transduction to look at the initial triggers of disease before remodeling.

Listen to this article's corresponding podcast at https://ajpheart.podbean.com/e/dcm-mutations-alter-intracellular-ca2-and-signaling/.

## INTRODUCTION

Dilated cardiomyopathy (DCM) is a disease of the myocardium characterized by dilatation of the left ventricle, impaired systolic function, evidence of cellular apoptosis, and interstitial fibrosis. Clinically, it is associated with chronic heart failure, sudden cardiac death, and cardioembolic stroke. DCM affects roughly 1 in 500 people globally (49). Approximately 30–40% of cases are found to be familial (19), making it one of the most commonly inherited conditions. Causative genes have substantial overlap with other cardiac conditions, such as arrhythmogenic right ventricular cardiomyopathy (ARVC) and hypertrophic cardiomyopathy (HCM); however, it is far more genetically heterogeneous than both of these, with over 30 potential disease genes characterized to date (53). These encode proteins involved in diverse cellular processes, including lamin A/C at the nuclear envelope (13), dystrophin, and δ-sarcoglycan in the dystroglycan complex (14, 50), muscle Lim protein (29), and Cypher/ZASP (52) involved in mechanosensation and Ca^2+^ handling proteins such as phospholamban (PLN) (43). In addition, mutations in sarcomeric proteins such as β-myosin heavy chain (β-MHC) (20), actin (33), cardiac troponin (TnT, TnI, and cTnC) (20, 28, 31), α-tropomyosin (α-TM) (32), and titin (18) make up a significant portion of the genetic burden. Our previous work examining an array of DCM-causing mutations in TnT [ΔK210 (40) and R131W, R141W, A172S, R205L and D270N (26)], TnI [K36Q and N185K (6)], and α-TM [E40K and E54K (27) and D230N (22)] has shown a uniform reduction in the Ca^2+^ sensitivity of actin-activated myosin ATPase activation. Furthermore, in vitro characterization using IAANS-labeled troponin C (TnC) to report myofilament Ca^2+^ affinity also showed a reduction in cooperative Ca^2+^ affinity of thin filaments containing DCM mutant troponin (Tn) or α-TM (38). The precise molecular mechanism of DCM pathogenesis as a result of mutations in the thin filament is, however, more complex. To date, five gene-targeted knock-in mouse models of DCM carrying thin-filament mutations have been characterized: TnT ΔK210 (10), R141W (36), and R134W (15); actin E99K (46); and αTM E54K (35). Each show similar macroscopic phenotypes but differing effects on contractility and Ca^2+^ transients in the isolated cardiomyocyte; for example, cardiomyocytes from the actin E99K mouse show reduced basal Ca^2+^, whereas TnT ΔK210 cardiomyocytes have increased systolic Ca^2+^ levels. This divergence in Ca^2+^-handling phenotype could be a combination of age/strain-dependent compensation, mutation severity, or subtle differences in primary mutant mechanism. In vitro work by the Marston laboratory has shown that DCM mutations blunt the Ca^2+^-desensitizing effect of β-adrenergic phosphorylation of TnI (25), suggesting a more complex primary driver of DCM pathology than simple myofilament Ca^2+^ desensitization. Further work by Davis et al. has suggested that manipulating Ca^2+^ sensitivity with artificially engineered TnC mutants can modulate the activation of Ca^2+^-dependent calcineurin/nuclear factor of activated T-cell (NFAT) signaling cascades while simultaneously inhibiting extracellular signal‐regulated kinase (ERK) signaling in the same cell (9). Additionally, a reduction in RAC-α serine/threonine protein kinase (Akt) activation in TnT R141W DCM transgenic mice has been observed (16). It is unclear whether this pathway, which is canonically linked to tyrosine kinase receptor/PIP_3_ pathway stimulation (48), is influenced by myofilament regulation or whether the changes in DCM are a consequence or primary driver of disease pathogenesis, given the role of Akt in cell survival. In this study we set out to investigate the functional effect of DCM-causing mutations in three separate thin-filament regulatory proteins: TnT R131W, TnI K36Q, and α-TM E40K, using our established adult guinea pig left ventricular cardiomyocyte model (39). We systematically assessed contractility, Ca^2+^ handling, and signaling to dissect the potential involvement of Ca^2+^ dysregulation in DCM disease pathogenesis. This study provides a direct comparison with our previous work on different mutations in the same genes that cause the phenotypically distinct HCM. As at the molecular level, the DCM and HCM subsets of mutations have opposing effects on contractile function, we postulated that thin-filament DCM mutations may cause distinct alterations in Ca^2+^ cycling and signaling compared with HCM mutations in the same model.

## METHODS

### 

#### Virus production.

Wild-type (WT) TnT, R131W TnT, WT TnI, and K36Q TnI, containing an NH_2_-terminal -DYKDDDDK- FLAG Tag and WT α-TM and E40K α-TM containing a COOH-terminal DYKDDDDK FLAG Tag, were cloned, recombined, and scaled using the AdEasy recombination system (Agilent Technologies), as previously described (24, 39). High titer adenovirus was purified using cesium chloride centrifugation, desalted using a 3-mL PD10 column, and mixed with an equal volume of 20% glycerol-80% FBS for cryostorage. The plaque forming unit (PFU) titer was estimated using the number of green cells infected by serial dilutions of purified virus in a plate containing ~2 × 10^6^ unmodified HEK293 cells over a 48-h period. Titers for R131W TnT, K36Q TnI, and E40K α-TM were ~4.8 × 10^10^, ~9.7 × 10^10^, and ~2.4 × 10^10^ PFU/mL, respectively; equivalent WT viral titers were calculated previously (39).

#### Cardiomyocyte isolation.

This investigation was approved by the Animal Welfare and Ethical Review Board at the University of Oxford and conforms to the UK Animals (Scientific Procedures) Act, 1986, incorporating Directive 2010/63/EU of the European Parliament. As previously described (39), 400-g adolescent male guinea pig hearts (to prevent confounding effects of hormonal cycle) were used to isolate left ventricular cardiomyocytes by direct collagenase (0.8 mg/mL) perfusion of the coronary artery. In total, 77 animals were used in this study. To isolate, a water-jacketed/temperature-regulated Langendorff apparatus was used under the control of a peristaltic pump to deliver preoxygenated isotonic Krebs isolation solution, consisting of (in mM) 130 NaCl, 23 4-(2-hydroxyethyl)-1-piperazineethanesulphonic acid (HEPES), 21 glucose, 20 taurine, 5 creatine, 5 MgCl, 5 Na pyruvate, 4.5 KCl, and 1 NaH_2_PO_3_ (pH 7.3 with NaOH), to the heart at 7 to 8 mL/min and 37°C for 5 min. The left ventricle was dissected and placed on a shaker for a further 10 min. Left ventricular cardiomyocytes (1.5 × 10^5^ cells/mL) were incubated in ACCITT_3_ culture medium (12) at 37°C and 5% CO_2_. Viral gene transduction was for 48 h following addition of ~1,000 multiplicity of infection (MOI) of recombinant adenovirus to the isolated cells, at which time functional analysis and/or biochemical experiments were performed. We found that as previously described (39), these experimental conditions gave ~90–100% of cardiomyocytes coexpressing GFP in addition to our DCM mutant or WT protein of interest. However, each cell was checked using and FTIC (495 nm/519 nm) filter set before all functional measurements. Any cell achieving lower that 25% or total fluorescence saturation was excluded from the study to ensure all single-cell measurements used cardiomyocytes with an even expression level of recombinant protein.

#### Simultaneous measurement of sarcomere shortening and Ca^2+^ transients.

Unloaded sarcomere shortening of isolated cardiomyocytes without fura-2 loading was measured at 1-Hz pacing. Acquisition relies on fast Fourier transform of a defined region of *z*-disk striations (acquired by phase-contrast imaging) at a sampling rate of 100 Hz. Unloaded sarcomere shortening measurements were also taken at 0.5 Hz following loading with 1 μM fura-2 for 5 min simultaneously with Ca^2+^ transients. This allowed diastolic Ca^2+^-transient measurements to be more stable at the slower pacing frequency; however, the effect of DCM mutations of contractility parameters was qualitatively comparable across both experimental conditions. Ca^2+^ transients were acquired using the ratio of fura-2 fluorescence emission at 360/380 nm at a switching rate of 1,000 Hz. All experiments were carried out at 37°C under constant 1 mL/min perfusion of a buffer containing (in mM) 150 NaCl, 10 HEPES, 7 glucose, 1 MgCl, 1 KCl, 0.3 NaH_2_PO_3_, 1.8 CaCl_2_ (pH 7.4 with NaOH) to avoid buffer electrolyte imbalance during electrical pacing. Finally, the ratio of fura-2 fluorescence signal was converted to intracellular Ca^2+^ concentration ([Ca^2+^]_i_) using a Ca^2+^ calibration kit with Mg^2+^ (Life Technologies), following the manufacturer’s protocol in a final buffer containing (in mM) 100 KCl, 1 MgCl_2_, and 30 MOPS (pH 7.2). All experiments were acquired using an IonOptix μstep system, transients were averaged and exported, and parameters were extracted using IonWizard v6.4.11. It was previously observed that the transduction of human flag tagged TnI and TnT does not significantly alter sarcomere shortening and Ca^2+^ transients compared with uninfected control cardiomyocytes from the same isolation. However, human flag tagged α-TM expression causes an increase in basal sarcomere length and relaxation time (39). We also see this alteration in this study and believe it is a result of the COOH-terminal flag tag disrupting the native end-to-end binding of α-TM. For this reason, it is essential that all DCM mutant comparisons are paired with a WT-transduced control for each protein expressed to make accurate observations and fully reflect the effect of each mutation tested.

#### Measurement of sarcoplasmic reticulum load, sodium-calcium exchanger, and SERCA2a activity.

Sarcoplasmic reticulum (SR) Ca^2+^ load was calculated under the condition given in the previous section by the direct application 10 mM caffeine (in Ca^2+^ perfusion buffer for 20 s) immediately after deactivation of pacing using a separate gravity perfusion system attached to a heated (37°C) perfusion pencil (Digitimer). It was ensured that switching of pacing and caffeine application was instantaneous, by using Clampex software (Axon Instruments) to control each event. Fractional Ca^2+^ release from the SR was calculated by the division of [Ca^2+^]_i_-transient amplitude preceding caffeine application by the SR [Ca^2+^]_i_ (given by the caffeine-transient amplitude). Sodium-calcium exchanger (NCX) activity was estimated by the τ-decay rate (τ_1_) following 10-mM caffeine application. Sarcoendoplasmic reticulum ATPase (SERCA2a) activity was calculated by the subtraction of the fast [Ca^2+^]_i_-transient τ_2_ decay rate, which gives the total intracellular Ca^2+^ reuptake, from the slower τ_1_ that was previously calculated (3).

#### Western blot analysis.

Western blot samples were prepared from 6 × 10^5^ infected cardiomyocytes in cell lysis buffer containing 20 mM imidazole (pH 7.0), 6 M urea, 0.4 M NaCl, 1 mM EDTA, 10% sucrose, 2 mM β-mercaptoethanol, 1% sodium dodecyl sulfate, 1% Triton X-100, 1 mM phenylmethanesulfonyl fluoride (PMSF), 1 mM Nα-*p*-tosyl-l-arginine methyl ester (TAME), and 1 mM tosyl phenylalanyl chloromethyl ketone (TLCK). To assess recombinant protein expression levels, cardiomyocytes underwent fluorescence-activated cell sorting (FACS; Jenner Inst., University of Oxford) to remove any uninfected cells from the suspension before sample preparation. This also served to suspend the cells in a defined volume of PBS per cell, thereby removing the need for a loading control. Samples were blotted using either mouse anti-FLAG-Tag (1/2,000) (Sigma), mouse anti-TnT (JLT clone; 1:2,000; Sigma), rabbit anti-TnI (1:3,000; Aviva Systems Biology), and mouse anti-α-TM (1:2,000; Sigma) overnight at 4°C, followed with a horseradish peroxidase (HRP)-conjugated bovine anti-mouse (Santa Cruz) or goat anti-rabbit IgG secondary antibody (1:4,000) at room temperature for 1 h.

For assessment of ERK, NFAT, and Akt activation, 6 × 10^5^ infected cardiomyocytes were chronically paced at 40 V, 0.5 Hz for 8 h using a C-Pace electrode array (IonOptix) or left as unpaced samples and prepared as above. Membranes were blotted using anti-ERK (1/3,000; Cell signaling) or anti-phospho-threonine 202/tyrosine 204-ERK (1/2,000; Cell Signaling), anti-NFAT-c3 (1 in 3,000; Santa Cruz), anti-phosho-serine-185 NFAT (1 in 3,000; Millipore), anti-Akt (1 in 500; Cell Signaling), or anti-phospho-serine 473 Akt (1 in 1,000; Cell Signaling), followed by goat anti-rabbit IgG-HRP secondary antibody (Promega). Luminescent bands were developed with ECL select substrate (GE Healthcare) for 5 min. Images were acquired using a Gel Doc imaging system (Bio-Rad). Antibodies were removed using 10 mL of Restore Western blot stripping solution (Thermo Scientific) at room temperature for 8–10 min. Membranes were reprobed with an anti-GAPDH (1:3,000; Sigma) that served as a loading control.

For assessment of Ca^2+^ handling proteins and contractile proteins 6 × 10^5^ infected cardiomyocytes were chronically paced at 10 V, 0.5 Hz for 8 h using a C-Pace electrode array (IonOptix) samples and prepared as above or, for PLN samples, were not denatured to allow the assessment of monomer and pentamer ratios. Membranes were blotted with anti-PLN (1 in 4,000; Badrilla), anti-phosho-Ser16 PLN (1 in 2,000; Upstate), anti-phospho-Thr-17 PLN (1 in 200; Santa Cruz), anti-TnI (1 in 1,000; Santa Cruz), anti-phospho-Ser 23/24 (1 in 500; Cell Signaling), anti-SERCA2a (1 in 1,000; Santa Cruz), anti-NCX (1 in 500; Alamone Laboratories), and L-type Ca^2+^ channel (1 in 500; Sigma), followed by either goat anti-rabbit (Promega), donkey anti-goat (Santa Cruz), or sheep anti-mouse (GE Healthcare) secondary antibodies. Luminescent bands were developed with Pico PLUS ECL reagent (Thermo Scientific) for 5 min. Images were acquired using a Gel Doc imaging system (Bio-Rad). Antibodies were removed using 10 mL of Restore Western blot stripping solution (Thermo Scientific) at room temperature for 8–10 min. Membranes were reprobed with an anti-GAPDH (1:3,000; Sigma) that served as a loading control. The intensity of the bands was determined using Image Laboratory software (Bio-Rad).

#### Immunolocalization.

Flag-tagged thin-filament protein localization was imaged in 48-h transduced cardiomyocytes immobilized in two-well Falcon-chambered cell-culture slides (Fisher scientific) precoated with 40 μg/mL laminin (BD Bioscience) at room temperature for 4 h. Cells were fixed with 4% methanol-free paraformaldehyde (TAAB) for 15 min at room temperature; cells were then skinned and slide blocked simultaneously for 1 h using PBS containing 0.1% Triton X-100 and 5% BSA. Cells were incubated overnight at 4°C with mouse anti-FLAG-tag (1:8; Sigma) and rabbit anti-α-actinin (Sigma; 1:500), followed by a further overnight incubation with goat anti-mouse IgG Alexa 564 (1:200; Life Technologies) and goat anti-rabbit IgG Alexa 633 (1:200; Life Technologies). Slides were mounted with slow-fade diamond with DAPI and a 1.5 thickness coverslip and visualized using a Leica TCS SP5 X confocal microscope with a 63× oil immersion objective.

For imaging of NFATc3 following chronic pacing (4 h), cells were immobilized, fixed, and skinned in a round coverslip-bottomed 35-mm culture dish (Matek) using the same conditions as above. Cells were stained with consecutive overnight incubations with first NFAT-c3 (1/50; Santa Cruz) primary and then goat anti-mouse IgG Alexa 568 (1/200; Life Technologies). Finally, the sides of the coverslip were removed using a heated metal scalpel and mounted and imaged as before.

#### Statistics.

All cardiomyocyte comparisons were from at least four separate cell isolations with a similar number of cells from each isolation analyzed, and *n* is the total number for each experiment of cells assessed for each WT/DCM mutant comparison. Any cell with sarcomere shortening or Ca^2+^, amplitudes, or kinetics exceeding two standard deviations from the mean upon analysis were excluded because of phenotypic heterogeneity arising in cultured primary cells. Groups were tested for normality (D’Agostino and Pearson test), and either an unpaired *t*-test or Mann-Whitney test was performed (GraphPad Prism) to compare WT to mutant for each protein expressed. For Western blot densitometry and immunolocalization, groups were tested for normality (D’Agostin and Pearson test), and either an unpaired *t*-test or Mann-Whitney test (GraphPad Prism).

## RESULTS

### 

#### DCM-causing mutations reduce contractile magnitude and slow contraction and relaxation.

**Fig. 1.**
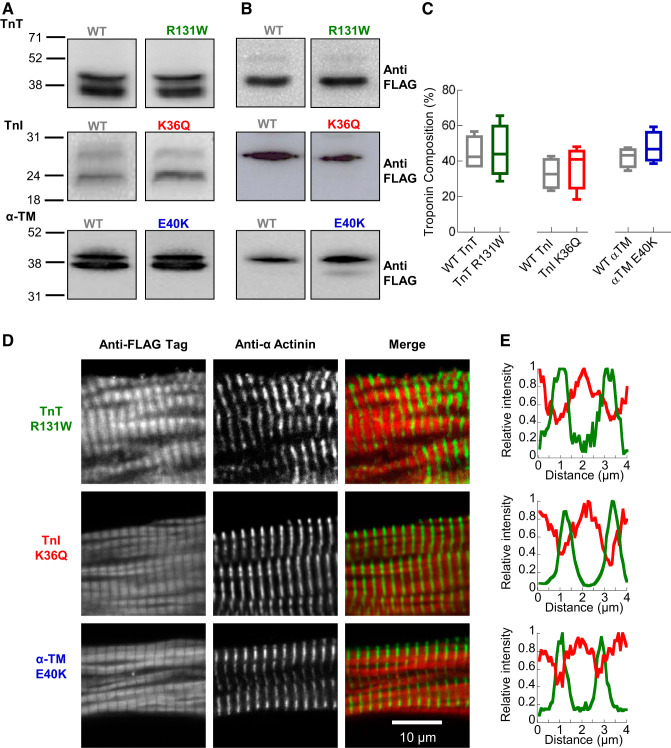
Expression and localization of adenovirally expressed FLAG tagged dilated cardiomyopathy (DCM) mutant protein in guinea pig left ventricular cardiomyocytes. WT, wild type; TnT, cardiac troponin T (R131W); TnI, cardiac troponin I (K36Q); α-TM, α-tropomyosin (E40K). *A*: relative protein expression of FLAG tagged human recombinant WT/R131W TnT, WT/K36Q TnI, and WT/E40K α-TM. The presence of an NH_2_- or COOH-terminal FLAG tag created a band shift in expressed protein to allow for direct comparison to endogenous guinea pig subunits. Blots were probed using anti-TnT, anti-TnI and anti-α-TM primary antibodies, respectively. *B*: same membrane probed using anti-FLAG tag primary antibody. The relative expression level of recombinant protein as a percentage of the total is plotted in *C* (*n* = 4). Box and whisker plot give the median (line), standard deviation (box) and maximum and minimum data spread (whiskers). Localization of adenovirally expressed FLAG tagged DCM mutant protein in guinea pig left ventricular cardiomyocytes is shown in *D*. Adenovirally expressed FLAG tagged protein was localized to the I band in laminin immobilized cardiomyocytes using an anti-FLAG tag primary antibody (with Alexa 568 secondary, red), with counterstain provided using an anti-α-actinin antibody (with Alexa 633 secondary, false colored green) to stain the *z* disks. Colocalization was confirmed in the intensity profile plots measured using ImageJ in *E*.

**Fig. 2.**
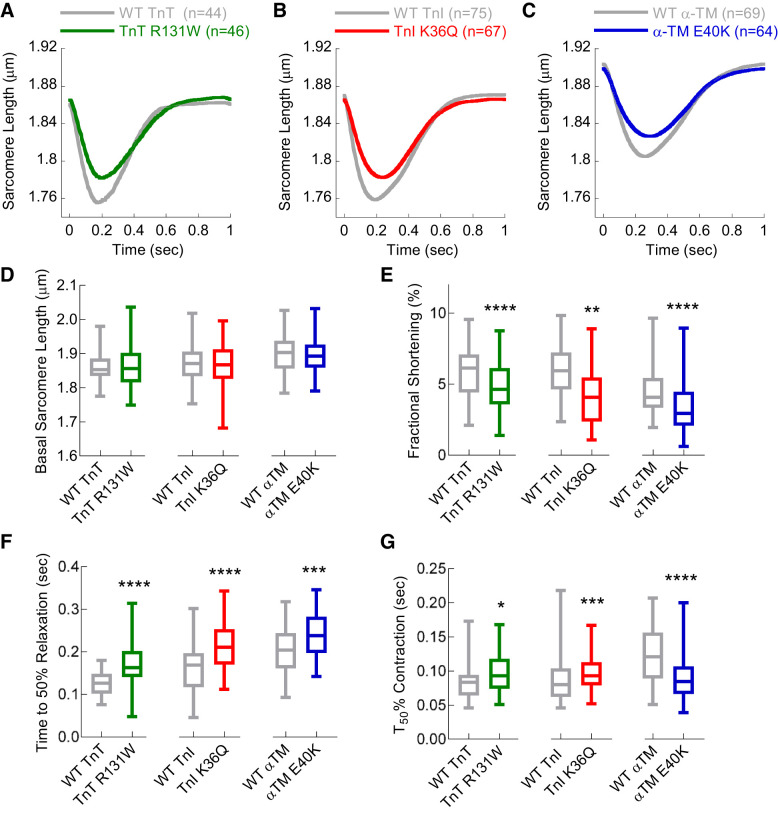
Unloaded sarcomere shortening measurements in adenovirally infected guinea pig left ventricular cardiomyocytes containing dilated cardiomyopathy (DCM)-causing mutations. WT, wild type; TnT, cardiac troponin T (R131W); TnI, cardiac troponin I (K36Q); α-TM, α-tropomyosin (E40K). *A–C*: unloaded sarcomere shortening measurements at 1 Hz pacing for TnT R131W, TnI K36Q, and α-TM E40K, respectively. *D–G*: basal sarcomere length, fractional shortening, time to 50% contraction, and time to 50% relaxation, respectively. Each curve was averaged from multiple cells (*n*) taken from at least 4 separate cell preparations; total *n* numbers are given in the legends of each plot. Box and whisker plots compare WT with DCM mutant for each gene tested and give the median (line), standard deviation (box), and maximum and minimum data spread (whiskers). All data were tested for normality (D’Agostino-Pearson), and significance values calculated using either Mann-Whitney or standard *t*-tests. *P < 0.05; **P < 0.01; ***P < 0.001; ****P < 0.0001.

We performed sarcomere shortening measurements in electrically paced (1 Hz) guinea pig left ventricular cardiomyocytes expressing either DCM mutant or corresponding WT human recombinant TnI, TnT, and α-TM. We first estimated the percent protein replacement of each of the six transductions by densitometry analysis of FACS-sorted cardiomyocytes to enrich the infected cell population in the resultant blots. We found that this enriched the functionally viable rod-shaped cardiomyocytes, as they were transduced and coexpressed GFP preferentially. Blotting using either anti-TnT, anti-TnI, or anti-α-TM (Fig. 1*A*) shows that the FLAG-tagged recombinant protein (Fig. 1*B*) is band shifted above endogenous protein in all cases. Incorporation of recombinant protein was 44.4 ± 4.2% for WT TnT, 45.5 ± 6.5% for TnT R131W, 32.9 ± 4.0% for WT TnI, 37.1 ± 5.6% for TnI K36Q, 42.2 ± 2.6% for WT α-TM, and 47.8 ± 3.7% for α-TM E40K (Fig. 1*C*), close to the 50% expected to be present in heterozygous patients (4). The viral infection levels (MOI) and transduction duration were carefully standardized by cell and PFU counts to maintain these levels for all subsequent functional experiments. The expected I-band incorporation of both WT (39) and mutant (Fig. 1, *D* and *E*) recombinant TnT, TnI, and α-TM was demonstrated by immunolocalization. Fast Fourier transformation of phase contrast-imaged *z*-disk striations gave an estimated basal sarcomere length of 1.86 ± 0.01, 1.88 ± 0.01, and 1.90 ± 0.01 µm for WT TnT, TnI, and α-TM, respectively; these were not significantly altered by the presence of DCM mutations in each protein (Fig. 2, *A*–*D*). DCM mutations, however, produced a consistent reduction in contractile magnitude of 19.8 ± 4.0, 32.6 ± 4.4, and 24.2 ± 6.2% for TnT R131W, TnI K36Q, and α-TM E40K, respectively (Fig. 2*E*). We also observe a slowing of relaxation kinetics with time to 50% (*T*_50_) of relaxation increasing by 44.2 ± 8.0, 55.2 ± 6.9, and 32.9 ± 6.4 ms for TnT R131W, TnI K36Q, and α-TM E40K, respectively (Fig. 2*F*). In contrast, there was not a consistent effect of the DCM mutants on contraction times: TnT R131W and TnI K36Q slowed *T*_50_ by 12.3 ± 4.2 and 9.9 ± 3.1 ms, respectively, whereas α-TM E40K has a 34.4 ± 4.1 ms faster *T*_50_ (Fig. 2*G*). Western blotting confirms that these changes in contractile performance are unlikely to be governed by changes in PKA-dependent phosphorylation of TnI, as the level of phospho-Ser 23/24 TnI is unaltered (Supplemental Fig. S1, *A* and *B*; all supplemental material may be found at https://doi.org/10.6084/m9.figshare.12161901).

#### DCM mutations reduce Ca^2+^-transient amplitude and prolong Ca^2+^ reuptake into the SR.

**Fig. 3.**
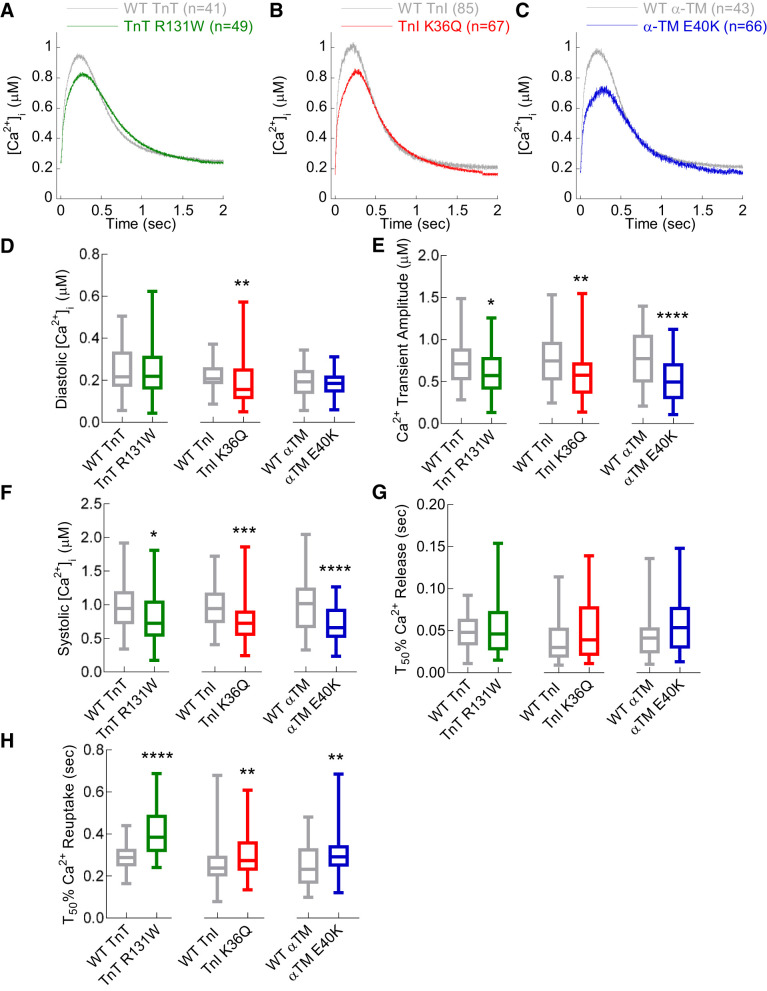
Ca^2+^-transient measurements in adenovirally infected guinea pig left ventricular cardiomyocytes containing dilated cardiomyopathy (DCM)-causing mutations. WT, wild type; TnT, cardiac troponin T (R131W); TnI, cardiac troponin I (K36Q); α-TM, α-tropomyosin (E40K). *A–C*: Ca^2+^-transient measurements at 0.5 Hz pacing, comparing WT to TnT R131W, TnI K36Q, and α-TM E40K, respectively. *D–H*: diastolic intracellular Ca^2+^ concentration ([Ca^2+^]_i_) systolic [Ca^2+^]_i_, Ca^2+^-transient amplitude, time to 50% (*T*_50_) sarcoplasmic reticulum (SR) Ca^2+^ release, and time to 50% SR Ca^2+^ reuptake, respectively. Each curve was averaged from multiple cells (*n*) taken from at least 4 separate cell preparations; total *n* numbers are given in the legends of each plot. Box and whisker plots compare WT with DCM mutant for each gene tested and give the median (line), standard deviation (box) and maximum and minimum data spread (whiskers). All data were tested for normality (D’Agostino-Pearson), and significance values calculated using either Mann-Whitney or standard *t*-tests. *P < 0.05; **P < 0.01; ***P < 0.001; ****P < 0.0001.

Having established that DCM mutations cause hypocontractility in our cardiomyocyte model, we next aimed to observe whether there was concomitant dysregulation in Ca^2+^ handling by measuring Ca^2+^ transients in cardiomyocytes loaded with fura-2 and paced at 0.5 Hz (Fig. 3, *A*–*C*). Diastolic Ca^2+^ levels remained unchanged in cardiomyocytes expressing TnT R131W and α-TM E40K compared with WT; however, TnI K36Q showed a 19.1 ± 5.7% reduction in basal Ca^2+^ levels (Fig. 3*D*). Ca^2+^-transient amplitudes were uniformly reduced (Fig. 3*E*) with a 19.7 ± 5.6, 19.9 ± 4.5, and 28.7 ± 3.5% reduction in systolic Ca^2+^ for TnT R131W, TnI K36Q, and α-TM E40K, respectively (Fig. 3*F*). There was no measurable change in Ca^2+^ release kinetics caused by the DCM mutations (Fig. 3*G*) despite altered contraction times observed for each mutant; this is potentially explained by *T*_50_ of Ca^2+^ release ~35–45 ms being difficult to measure precisely due to technical limitations and recorded values having high variance. However, in parallel with observations on relaxation times, the *T*_50_ of Ca^2+^ reuptake was significantly prolonged by 115.0 ± 14.7, 45 ± 13.9, and 57.6 ± 14.1 ms for TnT R131W, TnI K36Q, and α-TM E40K, respectively (Fig. 3*H*). Unloaded sarcomere shortening measurements were also acquired simultaneously with Ca^2+^ transients in cardiomyocytes loaded with 1 μM fura-2 paced at 0.5 Hz to compare the effects of the DCM mutations on sarcomere shortening measured in the absence of fura-2 at 1 Hz, discussed above (Supplemental Fig. S2, *A–C*). We found that fura-2 loading increased diastolic sarcomere length (Supplemental Fig. S2*D*), reduced fractional shortening (Supplemental Fig. S2*E*), and lengthened contraction (Supplemental Fig. S2*F*) and relaxation time (Supplemental Fig. S2*G*) when compared with the same contractile parameters from unloaded cardiomyocytes in Fig. 2. This is presumably due to Ca^2+^ buffering of the chemical Ca^2+^ dye, which has a *K*_d_ of ~150 μM and has been shown to accumulate to ~100 μM levels in various cell types (45). However, it was found that qualitatively, the effects of all three DCM mutants altered fractional shortening, contraction, and relaxation in the same way; we are therefore able to draw conclusions and assess the interaction between the effect of mutations on contractility and Ca^2+^, free of the bias of fura-2-induced contractile impairment in this study.

#### DCM mutations cause SR Ca^2+^ overload, increased NCX activity with compensatory-reduced SERCA2a activity.

**Fig. 4.**
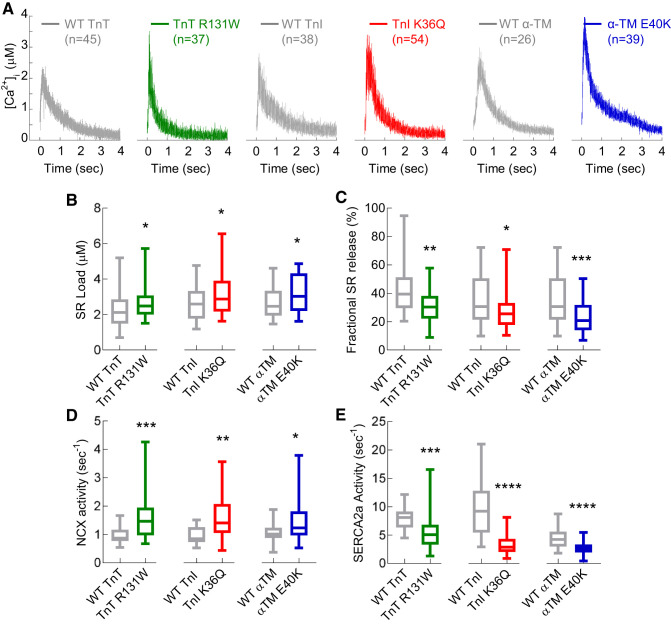
Dilated cardiomyopathy (DCM) mutant-infected cardiomyocytes have increased sarcoplasmic reticulum (SR) load and sodium-calcium exchanger (NCX) activity with reduced fractional SR Ca^2+^ release and sarco(endo)plasmic reticulum Ca^2+^ ATPase (SERCA2) activity. WT, wild type; TnT, cardiac troponin T (R131W); TnI, cardiac troponin I (K36Q); α-TM, α-tropomyosin (E40K). *A*: representative caffeine-induced intracellular Ca^2+^ concentration ([Ca^2+^]_i_) transients generated by the direct perfusion of 10 mM caffeine after a 5-s pause of pacing for WT, TnT, TnT R131W, WT TnI, TnI K36Q, WT α-TM, and α-TM E40K-transduced cardiomyocytes, respectively. Box and whisker plots show the SR load (*B*), fractional release of Ca^2+^ (calculated from the caffeine-transient amplitudes in *B* subtracted from the preceding Ca^2+^-transient amplitude shown in Fig. 2*F* (*C*). NCX activity was measured from the τ-decay rates of caffeine induced Ca^2+^ transients (*D*). SERCA2a activity was calculated by the subtraction of τ-decay constants derived from Fig. 2*H* and caffeine-transient (NCX) τ-decay rates from *D* (*E*). Box and whisker plots compare WT with DCM mutant for each gene tested and give the median (line), standard deviation (box), and maximum and minimum data spread (whiskers): All data were tested for normality (D’Agostino-Pearson), and significance values calculated using either Mann-Whitney or standard *t*-tests. *P < 0.05; **P < 0.01; ***P < 0.001; ****P < 0.0001.

**Fig. 5.**
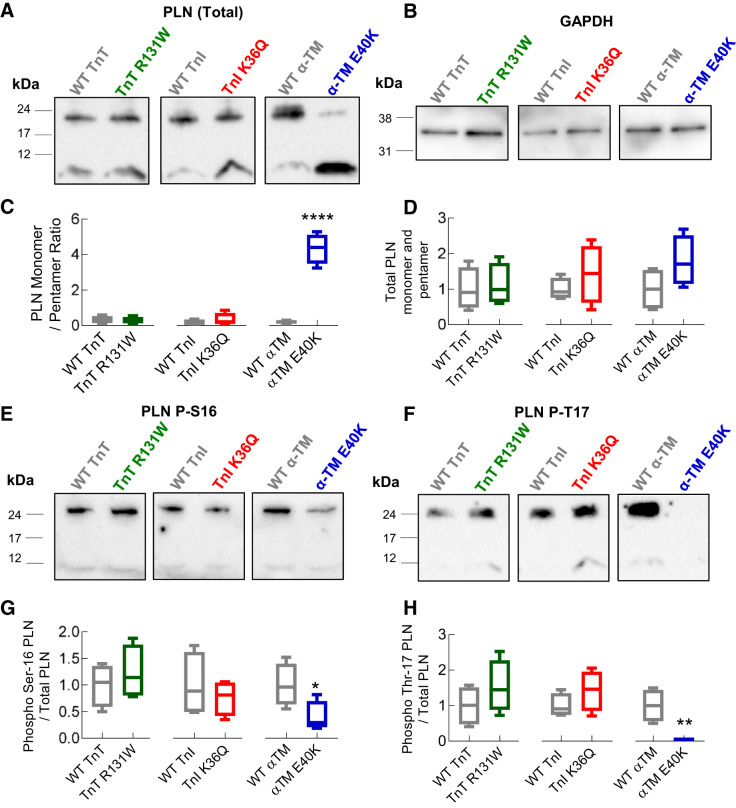
The ratio of monomeric vs. pentameric phospholamban (PLN) is increased by α-tropomyosin (α-TM; E40K)-dilated cardiomyopathy (DCM)-mutant protein expression following chronic pacing. WT, wild type; TnT, cardiac troponin T (R131W); TnI, cardiac troponin I (K36Q). Left ventricular cardiomyocytes were paced for 8 h at 0.5 Hz and 10 V at 37°C. Western blotting of PLN under nondenaturing conditions indicates that the total PLN levels are unchanged (*A* and *D*). When normalized to GAPDH loading controls (*B*), however, presence of the DCM mutation E40K in α-TM causes significant increase of the monomeric protein levels (*C*), driven by a reduction in PKA-dependent phosphorylation of serine-16 (*E* and *G*) and a profound reduction in CAMKII-dependent phosphorylation of threonine-17 when normalized to total PLN levels (*F* and *H*). Box and whisker plots give the median (line), standard deviation (box), and maximum and minimum data spread (whiskers) to compare DCM mutant to WT for each gene tested, and significance value was calculated using a Mann-Whitney test. *P < 0.05; **P < 0.01; ****P < 0.0001; *n* = 4 cell isolations for each group.

Ca^2+^ transients obtained following direct spritz with 10 mM caffeine highlight alterations to SR Ca^2+^ and the activities of the Ca^2+^ handling proteins NCX and sarco(endo)plasmic reticulum Ca^2+^ ATPase (SERCA2a) (Fig. 4*A*). SR Ca^2+^ load, as assessed by the amplitude of the caffeine-induced Ca^2+^ transients, was consistently higher by 24.0 ± 7.2, 22.7 ± 6.3, and 21.2 ± 6.4% in TnT R131W, TnI K36Q, and α-TM E40K cells, respectively, compared with WT (Fig. 4*B*). Combined with reduced amplitude of the field-stimulated Ca^2+^ transient, this resulted in a significantly smaller fractional release of Ca^2+^ from the SR (Fig. 4*C*). The rate of Ca^2+^ extrusion from the cytoplasm via the NCX was substantially increased by 0.75 ± 0.20, 0.61 ± 0.11, and 0.46 ± 0.13 s^−1^ in TnT R131W, TnI K36Q, and α-TM E40K, respectively (Fig. 4*D*). By contrast, SERCA2a activity was significantly reduced with all three mutations by 2.36 ± 0.68, 6.50 ± 0.24, and 1.64 ± 0.18 s^−1^ for TnT R131W, TnI K36Q, and α-TM E40K, respectively (Fig. 4*E*). This is in addition to increased SR load and therefore may represent a compensatory response. Western blotting confirmed that total levels of Ca^2+^ handing proteins, SERCA2a, NCX, and L-Type Ca^2+^ channel were unchanged (Supplemental Fig. S1, *C–F*). However, the E40K α-TM mutant, but not TnT R131W and TnI K36Q, showed a concordant stabilization of PLN monomer (Fig. 5, *A*–*D*), driven by a 60.1 ± 12.4% reduction in serine-16 phosphorylation (Fig. 5, *E* and *G*) and a 98.0 ± 2.3% reduction in threonine-17 phosphorylation (Fig. 5, *F* and *H*).

#### DCM mutations activate calcineurin-dependent and Akt signaling but have no effect on ERK activation.

**Fig. 6.**
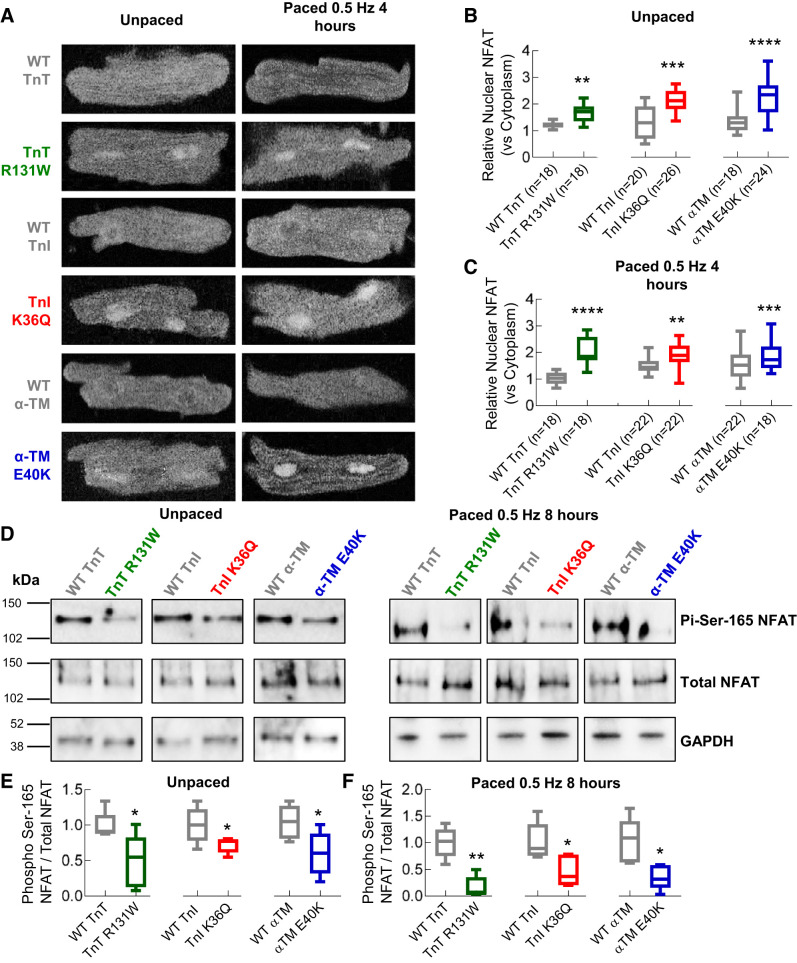
Dilated cardiomyopathy (DCM) mutant-infected cardiomyocytes have pacing-independent, dephosphorylation with concordant increased nuclear factor of activated T cells (NFAT) nuclear localization. WT, wild type; TnT, cardiac troponin T (R131W); TnI, cardiac troponin I (K36Q); α-TM, α-tropomyosin (E40K). *A*: representative cropped immunofluorescence images of single cardiomyocytes using anti-NFAT-C3 antibody. Individual panels compare DCM mutant to WT infected cardiomyocytes either unpaced or paced at 0.5 Hz for 4 h. The relative nuclear intensity of NFAT (colocalized with DAPI) (vs. cytoplasmic) is calculated using ImageJ, to compare WT to mutant cardiomyocytes for unpaced (*B*) and paced (*C*) cells, respectively. Concordant NFAT dephosphorylation is seen in Western blots comparing unpaced and paced, DCM and WT-transduced cardiomyocyte lysates, were developed with anti-NFAT, Anti-phospho-Ser-165 NFAT or anti-GAPDH antibody. Representative blots are shown in *D*. Box and whisker plots (*E* and *F*) compare DCM mutant to WT for each gene tested and for unpaced or paced cardiomyocytes, respectively. All box and whisker plots give the median (line), standard deviation (box), and maximum and minimum data spread (whiskers). All data were tested for normality (D’Agostino-Pearson) and significance values calculated using Mann-Whitney tests. *P < 0.05; **P < 0.01; ***P < 0.001; ****P < 0.0001; *n* = 5 blots for each group.

We observed the nuclear translocation of the transcription factor NFATc3 by coimmunolocalization with DAPI (Supplemental Fig. S3) in cells transduced with DCM-causing mutations, whereas there was no evidence of NFATc3 in the nucleus compared with cytoplasmic background in WT-transduced cells (Fig. 6*A*). This was independent of whether cardiomyocytes were unpaced (Fig. 6*B*) or paced for 4 h (Fig. 6*C*). We also showed that NFAT nuclear translocation is driven by significant dephosphorylation of Ser-165 (Fig. 6*D*) in both unpaced (Fig. 6*E*) and paced cells (Fig. 6*F*).

**Fig. 7.**
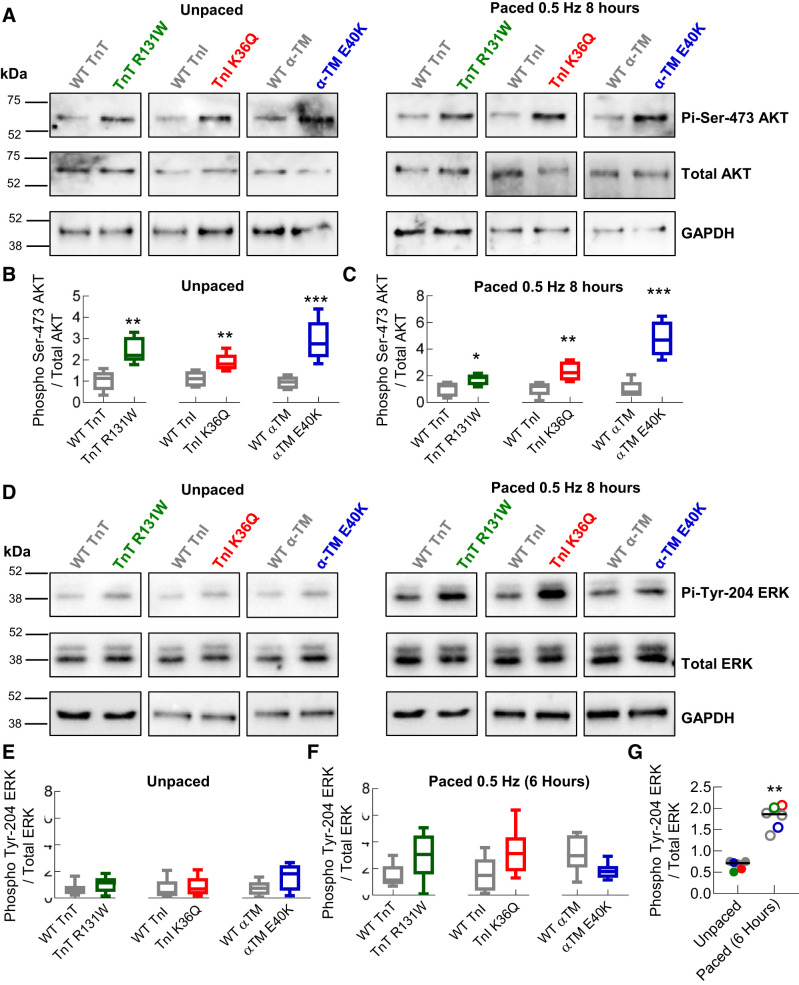
Dilated cardiomyopathy (DCM) mutant cardiomyocytes increase pacing independent Akt phosphorylation with no change to ERK phosphorylation compared with wild type (WT). TnT, cardiac troponin T (R131W); TnI, cardiac troponin I (K36Q); α-TM, α-tropomyosin (E40K). Western blots comparing unpaced and paced (at 0.5 Hz for 8 h), DCM and WT-transduced cardiomyocyte lysates, were developed following incubation with anti-Akt, anti-phospho-Ser-473 Akt, anti-ERK, anti-Tyr-204 ERK or anti-GAPDH antibody. Representative blots for Akt and ERK are shown in (*A* and *D*), respectively. Box and whisker plots show the relative densitometry analysis of phosphorylated vs. total protein, give the median (line), standard deviation (box), and maximum and minimum data spread (whiskers) to compare DCM mutant to WT for each gene tested and for either unpaced (*B* and *E*) or paced (*C* and *F*) cardiomyocytes. The dot plot (*G*) shows the average phosphorylation level of ERK in each group to compare unpaced to paced cardiomyocytes; black line gives the mean average. Significance values were calculated using a Mann-Whitney test. *P < 0.05; **P < 0.01; ***P < 0.001; *n* = 5–8 blots for each group.

Short-term expression of DCM mutations also appears to increase the phosphorylation level Akt at Ser-473 irrespective of pacing (Fig. 7, *A*–*C*). This suggests both this pathway and calcineurin/NFAT activation are driven directly by the expression of DCM mutant troponin and that the mutations affect the distribution of Ca^2+^ in a resting cardiomyocyte independent of Ca^2+^ cycling.

It has been suggested that NFATc3 translocation is a hallmark of both HCM and DCM, whereas ERK activation is increased in HCM and reduced in DCM, thereby differentiating between the distinct remodeling observed clinically (9). In comparison with unpaced cells, Western blots show robust activation of ERK phosphorylation at Tyr-204 upon chronic pacing at 0.5 Hz for 8 h in all groups (Fig. 6, *D*–*G*). However, no change was observed between DCM-causing mutations and their respective WT-transduced controls in both unpaced (Fig. 7, *D* and *E*) and paced (Fig. 7, *D* and *F*) cardiomyocytes.

## DISCUSSION

Here we describe the effects of three human DCM mutant proteins (TnT R131W, TnI K36Q, and αTM E40K) on cardiomyocyte contractility, Ca^2+^ handling, and signaling. After 48 h of transgene expression using adenoviral transduction, we observed reduced contractility and peak systolic Ca^2+^, along with an increase in NCX activity and SR Ca^2+^ load. Additionally, NFATc3 nuclear translocation was observed and Akt activation was increased in mutant cardiomyocytes compared with cells expressing the corresponding WT protein. These changes were consistent for each of the mutations in three different disease genes. This allows us to suggest that these changes are among the primary mechanisms of disease pathogenesis, in a model free of secondary compensatory molecular alterations.

In this study we have modeled DCM using cultured guinea pig left ventricular cardiomyocytes. We acknowledge that some dedifferentiation occurs in our cells and the use of FLAG-tagged exogenous protein expression to model disease can affect myocyte function, particularly in α-TM where the tag could potentially affect end-to-end binding; however, previous studies have shown that the effect on function is small and consistent (39), thus validating careful pairwise comparisons between WT and mutant settings. Furthermore, we believe guinea pig cardiomyocytes more accurately reflect the ion channel and sarcomeric protein milieu of human cells than do mouse cardiomyocytes (39). Murine ventricular cardiomyocytes express primarily fast α-myosin, whereas slow β-myosin predominates in both human and guinea pig ventricles (37); this switch of myofilament protein isoform is known to significantly modify findings and interpretations of molecular mechanism when testing DCM-causing mutations in TnT (15). Furthermore, NCX activity contributes ~30% of the total Ca^2+^ transient in our model and in humans, whereas murine cardiomyocytes rely almost exclusively on Ca^2+^-induced Ca^2+^ release (CICR) from the SR (2). Recordings of murine action potential also display marked differences, suggesting significant differences in ion channel isoform and activities compared with human and guinea pig hearts, which display similar hallmarks to one another (41). The choice of model appears to be validated in our study, where significant novel alterations to Ca^2+^ handling in the presence of DCM-causing mutations were uncovered, which had not been previously seen in established mouse models (10, 36, 46). To date, similar Ca^2+^-transient alterations have only been noted in a study using patient derived-induced pluripotent stem cell cardiomyocytes (iPSC) containing a TnT R173W DCM-causing mutation (47).

Some studies on transgenic mice with Ca^2+^-desensitizing mutations, however, have observed the opposite result with respect to Ca^2+^ transients as previously discussed. For example, work on a murine model that artificially manipulates troponin C (TnC) Ca^2+^ affinity using an I61Q mutation found increased Ca^2+^-transient amplitude (9). The opposing findings of our study suggest that NCX is activated and more readily reversed to remove excess Ca^2+^ in systole due to lower coordination of Ca^2+^ by the myofilament containing DCM mutations. The NCX is more highly expressed in guinea pig cardiomyocytes compared with mice (2, 11) and is therefore able to remove a greater amount of excess Ca^2+^ not bound to the myofilament in our model and thereby provide a tractable mechanism by which systolic Ca^2+^ is reduced compared with murine models (9, 30). Furthermore, the mouse model findings could also represent a compensation of this phenomenon due to long-term transgene expression or a model-specific observation that may not be recapitulated in other species.

We cannot rule out the influence of impairment to Ca^2+^ release; DCM mutant cells have a paradoxical increase in SR Ca^2+^ content but a lower fractional release in combination with the maintenance of basal Ca^2+^ levels. SERA2a activity is also clearly suppressed in our DCM model compared with WT, which may be a direct effect of a concentration gradient driven by lower cytoplasmic Ca^2+^ in systole and higher SR Ca^2+^ content.

Prolonged contraction and relaxation appear paired with an increase in the time to Ca^2+^ reuptake (likely caused by lower SERCA2a activity). Again, this is at odds with the findings in short-term expression models used in Davis et al. (9), where Ca^2+^ transients returned to baseline more quickly when the myofilament was directly desensitized and suggests that DCM mutations in the thin filament, which are spatially remote from the regulatory binding site of TnC, drive disease pathogenesis in a mechanistically more complex manner than first thought.

We have previously shown the presence of DCM-causing mutations (including TnT R131W, and TnI K36Q) in the isolated troponin complex do not directly alter Ca^2+^ affinity of the regulatory binding site of TnC. Instead, these mutations require the reconstitution of thin filaments, and hence cooperative communication from multiple troponins along the actin filament, to give a net reduction in Ca^2+^ affinity (6, 38). The mechanism by which these mutations can reduce Ca^2+^ affinity, which might be expected to reduce the dissociation constant of Ca^2+^ release from the myofilament, but prolong Ca^2+^ reuptake is unclear; we are able to suggest two potential pathways to explain this: first, Ca^2+^ release from the myofilament is quicker; however, the return of Ca^2+^ to the SR is prolonged by reduced SERCA2a activity. Alternatively, there could be more complex cooperative protein interactions in intact sarcomeres containing the mutations, for example, via myosin-binding protein C, which is known to interact with the thin filament (44) and affect the recently characterized superrelaxed state of myosin (1) or the interaction of myofilament phosphorylation status and net contractile function.

Each mutation appears to show subtle differences with respect to their knock-on effect to contractile function. For example, TnT R131W and TnI K36Q increase contraction speed compared with αTM E40K where relaxation is slowed; this could reflect alterations in the precise molecular mechanisms that underlie contractile impairment, for example, the position of each mutation with respect to the underlying actin monomer. Memo et al. (25) provided further evidence for mechanistic complexity. Assessing thin-filament DCM mutations using in vitro motility assays, they showed that DCM mutations did not decrease the Ca^2+^ sensitivity of thin filaments directly, rather they blunted of the Ca^2+^-sensitizing effect of PKA-dependent phosphorylation. How this phenomenon relates to Ca^2+^-transient amplitude reduction and to TnC Ca^2+^ buffering in our model would need to be investigated more fully.

We observe an acute, pacing-independent activation of the calcineurin-NFAT and Akt pathways. The fact that both occur in the absence of pacing is novel and unexpected. This suggests that the simple presence of DCM-causing thin-filament mutations is activating the calcineurin/NFAT and Akt pathways. We hypothesize that DCM mutant proteins could passively alter basal Ca^2+^ levels in certain subcellular pools when the cell is at rest, although there is no significant change to initial overall Ca^2+^ baselines in our recordings. There may even be potential for mutant troponin to interact directly with signaling molecules and act as cofactors for their activation. There is some evidence for altered myofilament troponin incorporation in induced pluripotent stem cell cardiomyocytes (iPSC) derived from patients with a ΔK210 TnT DCM mutation, where mutant protein was also detected in the nucleus in one third of the cell population (55).

We find that Akt phosphorylation is increased in our short-term DCM expression model, which is contrary to work in TnT R141W transgenic mice which have shown that Akt is dephosphorylated (16). Furthermore, increased Akt phosphorylation has been recently shown in iPSCs-expressing HCM mutant myosin (7), whereas constitutive activation of Akt also causes massive ventricular hypertrophy in mice (23). However, there is some evidence that Akt phosphorylation is controlled temporally; for example, a study of exercise-induced hypertrophy show that mice have short-term dephosphorylation of Akt at 1 wk of treatment that is followed by a marked increase in phosphorylation at 4 wk (17). It is possible there is a similar response of Akt to DCM mutations, where Akt is initially phosphorylated to assist cell survival, followed by a chronic reduction in Akt activation as remodeling occurs.

This study has revealed multiple novel changes in Ca^2+^ homeostasis and signaling in DCM using these specific mutations and this particular cellular background. We cannot exclude the possibility of other synergistic drivers of DCM disease pathogenesis. For example, it is well characterized that that energetic compromise is a robust feature of DCM and heart failure in general (8). It has also been shown that disrupted signaling between mitochondrial Ca^2+^ and energetic and apoptotic signaling pathways is also a feature of heart failure and DCM (21, 42).

We have previously highlighted that the DCM-causing mutations differ functionally at the molecular level from HCM-causing mutations in the same gene (26, 38, 40). Here we are able to compare the cellular effect of DCM mutations to our previously published work on HCM-causing mutations from the same genes (39). We found that the HCM mutants TnT R92Q, TnI R145G, and α-TM D175N all increased myofilament Ca^2+^ affinity which increases the in situ buffering capacity of the myofilament for Ca^2+^ during the contractile cycle. The resultant changes to Ca^2+^ handling include hypercontractility-increased diastolic Ca^2+^, increased SERCA2a activity, ryanodine receptor leak, and activation of ERK signaling. The DCM mutants tested in this study are hypocontractile, decrease systolic Ca^2+^, reduce SERCA2a activity, do not alter ERK phosphorylation, and thereby could represent opposing drivers of the phenotypically distinct macroscopic remodeling in DCM. However, there are also some similarities between HCM and DCM cellular remodeling (54), which may be driven by common alterations to Ca^2+^ handling and contractility, such as prolonged relaxation, Ca^2+^ reuptake, and increased SR load. Such alterations may drive NFAT activation in the presence of HCM- or DCM-causing mutations, hence driving some aspects of disease pathogenesis in both disease states.

This study shows why simple small molecule myofilament Ca^2+^ sensitizers/activators such as levosimendan (5, 34) or omecamtiv mecarbil (51) may not represent the ideal treatment strategy for patients with myofilament DCM, since the underlying pathogenesis of the disease is more complex than first believed.

## GRANTS

This work was funded by British Heart Foundation (BHF) Programme Grant RG/12/16/29939, BHF Centre of Research Excellence Oxford Grant RE/13/1/30181, and BHF Grants PG/18/68/33883 (to A.J.S.) and CH/1992001/6764 (to P.R.).

## DISCLOSURES

No conflicts of interest, financial or otherwise, are declared by the authors.

## AUTHOR CONTRIBUTIONS

P.R., Y.H.Z., B.C., H.W., and C.R. conceived and designed research; P.R., A.J.S., S.P., M.M., S.N.R., and Y.H.Z. performed experiments; P.R., A.J.S., S.P., and S.N.R. analyzed data; P.R., A.J.S., S.N.R., and C.R. interpreted results of experiments; P.R. prepared figures; P.R. drafted manuscript; P.R., A.J.S., B.C., and C.R. edited and revised manuscript; P.R., Y.H.Z., B.C., H.W., and C.R. approved final version of manuscript.
